# Impact of COVID-19 on Dutch General Practitioner Prenatal Primary Care: Retrospective, Observational Cohort Study Using an Interrupted Time-Series Approach

**DOI:** 10.2196/64831

**Published:** 2025-05-27

**Authors:** Wikje Berends-Hoekstra, Maarten Homburg, Anke Oenema, Matthijs Simeon Berends, Lilian Peters

**Affiliations:** 1 Department of Primary and Long-term Care, University of Groningen, University Medical Center Groningen, Home Post Code FA21, PO Box 196, Groningen, 9700 AD, The Netherlands, 31 625716156; 2 Department of Midwifery Science, Vrije Universiteit Amsterdam, Amsterdam University Medical Centre, Amsterdam, The Netherlands; 3 Midwifery Academy Amsterdam Groningen, Inholland University of Applied Sciences, Amsterdam, The Netherlands; 4 Department of Health Psychology, Open Universiteit, Heerlen, The Netherlands; 5 Department of Medical Microbiology and Infection Prevention, University of Groningen, University Medical Center Groningen, Groningen, The Netherlands; 6 Department of Medical Epidemiology, Certe Foundation, Groningen, The Netherlands

**Keywords:** pregnant women, COVID-19 pandemic, general practitioner, GP, health care–seeking behavior, interrupted time-series analysis, health policy, primary care

## Abstract

**Background:**

The COVID-19 pandemic significantly impacted primary health care–seeking behavior of the general population. The extent to which health care–seeking behavior of pregnant women in general practitioner (GP) care was affected remains largely unknown. The unique health care needs of pregnant women necessitate regular monitoring and care to ensure the well-being of expectant mothers, fetuses, and neonates, as timely interventions and screenings can profoundly influence the long-term health outcomes. Understanding how pandemic-related changes have influenced pregnant women’s primary health care–seeking behavior is essential for developing targeted interventions and informing policy decisions to improve health outcomes for expectant mothers, fetuses, and neonates, both during public health emergencies and in routine health care settings.

**Objective:**

This study aims to examine the impact of different COVID-19 pandemic phases on health care–seeking behavior among pregnant women in Dutch GP practices throughout 2020 and 2021. By analyzing clinical electronic health record (EHR) GP data, we aim to evaluate the health care consumption, occurrence of pregnancy-relevant symptoms and diagnoses, and types of contact (ie, regular consultations, phone consultations, home visits, and digital consultations) during different pandemic phases.

**Methods:**

Using a retrospective cohort design, EHRs of selected pregnant women from 3 Dutch GP networks between 2019 and 2021 were analyzed, comparing 6 pandemic phases divided into 13 subphases with a prepandemic phase. Contact rates were analyzed by interrupted time-series analyses, pregnancy-relevant symptoms, and diagnoses by comparing the frequency of pregnancy-relevant International Classification of Primary Care (ICPC) code registrations and type of contact by descriptive statistics.

**Results:**

In total, 10,985 pregnant women were included, yielding 39,023 patient-GP contacts. Contact rates fluctuated significantly across pandemic phases, with the sharpest declines at the onset and the end of the pandemic. Pregnancy-relevant symptoms and diagnosis in the category related to pregnancy showed the highest variability across the pandemic phases, such as an increase in the frequency of health care consumption concerning gestational diabetes mellitus and nausea or vomiting of pregnancy. Detailed statistical results are reported in the main text. Contacts for symptoms and diagnosis like digestive or urinary tract problems did not fluctuate across the pandemic phases. The number of physical contacts decreased, while telephone contacts increased.

**Conclusions:**

By analyzing EHR data from over 10,000 pregnant women, this study highlights the pandemic’s impact on pregnant women’s GP health care–seeking behavior, including declining health care consumption trends during the initial and end phases of the pandemic (2020‐2021). The observed increase in GDM and its potential long-term effects underscore the need for enhanced public health strategies within GP practices, ensuring continuous access to prenatal care and striving for improved outcomes of expectant mothers, their fetuses, and neonates during times of pandemics and in routine health care settings.

## Introduction

The COVID-19 pandemic has caused profound changes in health care–seeking behavior globally, especially within primary general practitioner (GP) care settings, where a significant decline in use was observed following the outbreak of the pandemic [1-5]. This decline in GP health care visits was shown to be driven by factors such as lockdown measures and heightened fears of viral transmission [1]. These shifts have resulted in reduced experienced accessibility and continuity of GP care, leading to postponed chronic care and increased interactions with unfamiliar health care providers [6].

Prenatal health care is managed collaboratively by midwives and obstetricians in secondary health care, and by midwives and GPs in primary health care. In the Netherlands, every citizen is registered with a GP who serves as a gatekeeper to secondary care [7]. Midwives and GPs typically oversee low-risk pregnancies in primary care, while obstetricians handle high-risk cases in secondary hospital care. GPs play a pivotal role in prenatal health care, providing comprehensive support that encompasses health promotion (eg, advices concerning diet and exercise in the case of diabetes mellitus, smoking cessation, and COVID-19 infection and vaccination), timely interventions for routine health care problems (eg, treatment of urine tract infections and mental health problems) and specialist referrals [8-10]. In addition, pregnant women can rely on GPs for certain medication needs (eg, antibiotics, thyroid medication, and mental health medication), especially since midwives do not have prescription authority in the Netherlands. A previous national study showed that before the pandemic, pregnant women typically consulted their GPs around 6 times on average during pregnancy and the postpartum period [10]. Common reported symptoms and diagnoses included intercurrent diseases, vomiting, urogenital problems, mental health issues, and work-related concerns [10,11]. Most pregnant women saw their GP, alongside their midwife or obstetrician, as an essential prenatal care provider, fostering long-term trusted health relationships with their registered GPs [10]. Therefore, GPs play an important role in providing additional prenatal health care for pregnant women.

Postponing or cancelling this important prenatal care may lead to negative consequences for maternal, fetal, or neonatal health [12-14]. Pregnant individuals are inherently vulnerable and susceptible to both physical and psychological illness, which may be exacerbated if the necessary prenatal GP care is not provided. Therefore, sustainable GP care for pregnant women, in addition to regular perinatal care, is needed for a robust public health care system [2].

Consequently, understanding the impact of the COVID-19 pandemic on health care–seeking behavior among pregnant women in GP practices is imperative. However, the extent to which the pandemic influenced pregnant women’s health care–seeking behavior within Dutch GP practices across different pandemic phases remains largely unknown. For that reason, this study aims to examine the impact of different COVID-19 pandemic phases on health care consumption among pregnant women in Dutch GP practices throughout 2020 and 2021. By analyzing routine clinical electronic health record (EHR) data registered by GPs, this study aims to evaluate health care consumption, the occurrence of pregnancy-relevant symptoms and diagnoses, and the types of contact (ie, regular consultations, phone calls, home visits, digital consultations) during different pandemic phases. It is important for informing policymakers, health care providers, and expectant mothers to understand how external factors such as a pandemic and associated restrictive measures influence health care–seeking behaviors among pregnant women. By providing these insights, this study ultimately aims to contribute to the improvement of prenatal GP care delivery, impacting public health in both pandemic and routine health care settings.

## Methods

### Study Design and Population

This retrospective observational cohort study used data retrieved from routinely registered clinical EHRs from GP practices from 2019 (prepandemic) to 2021 (pandemic). Data from 3 regional Dutch GP research networks were used: (1) the northern Academic General Practitioner Development Network (Academisch Huisartsen Ontwikkel Netwerk, AHON), including 59 GP practices managed by the University Medical Center Groningen (UMCG) [15]; (2) the eastern Family Medicine Network including 6 practices managed by Radboud University Medical Centre Nijmegen [16]; and (3) the southern Research Network Family Medicine Maastricht, including 28 practices managed by Maastricht University Medical Centre [17]. The network populations are a good reflection of the population in these regions [15,17,18]. The dataset included anonymized EHR data from approximately 410,000 patients, encompassing patients’ medical history, clinical findings, diagnoses, types of contact, and demographic characteristics [1].

In the Netherlands, GPs are required to assign an International Classification of Primary Care (ICPC) code to each patient contact [19]. Contacts were defined as either a physical consultation (at the clinic or at home), a telephone consultation, or any type of digital contact between a patient and a GP or practice nurse, with registration of a free text note (physician’s note) and at least 1 ICPC code.

### Selection of Pregnant Women

Women in the reproductive age (20‐45 y) with a confirmed pregnancy status in their EHR were selected for inclusion in the study. To select pregnant women, all EHR records of women of reproductive age were screened to determine pregnancy status during the studied period, using 3 methods. First, pregnancy status was determined based on the registration of at least one of the 24 pregnancy and childbearing related ICPC codes, such as W78 (“pregnancy, confirmed”), see Multimedia Appendix 1. Second, to identify pregnant and postpartum women without pregnancy-relevant ICPC codes, physician’s notes (free text notes) were screened for text patterns indicating pregnancy, using a regular expression (see Multimedia Appendix 2). To enhance validity, this regular expression was reviewed by a data scientist. Third, physician’s notes of contacts of potentially pregnant women selected by the aforementioned methods were manually assessed by the study team (WB-H and MH) to confirm pregnancy status (see Multimedia Appendix 3). Only contacts where pregnancy status was confirmed at the time of the contact were included irrespective of their pregnancy duration.

Women miscarrying were also retrospectively labelled as being pregnant. If neither of these 3 methods were irrefutable about pregnancy status, women were excluded. Registrations of the ICPC code W90 (“normal delivery liveborn”) were excluded for data integrity reasons, as some of these were identified as false registrations from previous pregnancies. Women were excluded when they had died during the study period, were deregistered from one of the included practices, had a reason for deregistration without deregistration date, had an unknown date of birth, were registered less than 3 months, or had missing data that hindered pseudonymization (eg, zip code). Only dates of weekdays were included. Weekend days, holidays, and consults outside of regular office hours were excluded from the analysis (see Multimedia Appendix 4).

### Outcome Variables

The primary outcome variables included pandemic health care consumption (ie, contact rates), pregnancy-relevant symptoms and diagnoses, and type of contact (ie, regular, phone, home visit, or digital contact), and were compared with the prepandemic baseline (2019). Pregnancy-related symptoms and diagnoses were determined by listing pregnancy-relevant ICPC codes that represented symptoms or diagnoses that commonly occur during pregnancy or require clinical attention in pregnant women. The selection was based on relevant ICPC codes from previous research [10], and supplemented with manual evaluation of all ICPC codes for their relevance in pregnancy. To validate the list of pregnancy-relevant ICPC codes, a general practitioner (MH) and a midwife evaluated the relevance of each code to pregnancy (see Multimedia Appendix 5). Contacts could involve one or more ICPC codes.

### Pandemic Phases and Subphases

Previous research showed that health care consumption fluctuated during the pandemic in response to both changes in COVID-19 incidence rates and the implementation or relaxation of restrictive measures in the general population [1]. Therefore, this study is conducted in accordance with a previously described framework that divides the pandemic into 6 main phases based on the restrictive measures mandated by the Dutch government [1,20]. These phases were subdivided into subphases based on turning points in the national COVID-19 incidence rates within each main phase (Table 1), using data from the National Institute for Public Health and Environment (Rijksinstituut voor Volksgezondheid en Milieu). In the general population, for example, health care consumption initially decreased in phase 1 and then increased again midway through phase 1 as incidence rates declined, leading people to gradually visit their GPs more often again [1].

**Table 1.** COVID-19 pandemic phases in the Netherlands based on infection rates and restrictive measures, and subphases based on highest and lowest infection rates.

Phase and subphase		Year	Week within year	Description of COVID-19 infection rates and containment measures
Phase 0				No confirmed COVID-19 infections in the Netherlands.
	Phase 0	2019	1‐52	
	Phase 0	2020	1‐8	
Phase 1				First wave of COVID-19 infections. First lockdown (ie, hand hygiene, social distancing, working at home, and schools, restaurants, amusement and industry closed). Highest infection rates: week 15.
	Phase 1a	2020	9‐13	
	Phase 1b	2020	14‐22	
Phase 2				Decrease in infection rates. Gradual relaxation of restrictive measures. Lowest infection rates: week 28.
	Phase 2a	2020	23‐28	
	Phase 2b	2020	29‐40	
Phase 3				Second wave of COVID-19 infections. Stricter containment measures, start of a partial lockdown followed by a strict lockdown (ie, curfew, schools, stores, and sport facilities closed). Highest infection rates: week 44 and 52.
	Phase 3a	2020	41‐44	
	Phase 3b	2020	45‐53	
	Phase 3c	2021	1‐3	
Phase 4		2021	4-17	Emergence of the Alpha variant (B1.1.7) of concern, further increase in infection rates, and continued lockdown measures. Highest infection rates: week 16.
Phase 5				Decrease in infection rates. Gradual opening-up of society and only minor restrictions. Lowest infection rates: week 26.
	Phase 5a	2021	18‐26	
	Phase 5b	2021	27‐43	
Phase 6				Epidemic rise with the Delta variant (B.1.617.2) of concern and a steep increase in infection rates. Lockdown measures reintroduced. Highest infection rates: week 48.
	Phase 6a	2021	44‐48	
	Phase 6b	2021	49‐52	

### Data Analysis

#### Overview

Descriptive statistics were performed to describe the study population characteristics for each studied year (2019, 2020, and 2021). This included the total number of included pregnant women per (pre)pandemic year and region, the total number of contacts, the mean number of contacts per patient with SD, the mean number of ICPC code registrations per contact, and the mean age with SDs. Statistical differences between the studied years were tested using a G-test for goodness-of-fit.

All statistical analyses with a *P*<.05 were considered significant. All data were analyzed using R (version 4.0.5; R Foundation for Statistical Computing).

#### Analysis of Health Care Consumption

Health care consumption was calculated by normalizing daily contact rates per 1000 registered pregnant patients. An interrupted time-series analysis was conducted using a segmented linear regression model to assess the impact of the COVID-19 incidence rates and societal measures on health care consumption during each pandemic subphase. For each (pre)pandemic subphase, a linear regression model was used, assuming a linear relationship between time and the contact rates within each subphase and fitted to generate a least squares regression line. Linearity was checked through visual inspection of the fitted models, while autocorrelation was assessed visually using autocorrelation plots. To enhance the stability of contact rates, a 3-week centered moving average was applied before modeling. Seasonality was accounted for by adding seasonal intercepts to the model, with spring as the baseline. For each subphase P, the linear regression model was described mathematically (Equation 1).


(1) $$Y_{T}\left(P\right)=\beta_{0}+\beta_{1}\cdot T+\beta_{2}\cdot S_{1}+\beta_{3}\cdot S_{3}+\beta_{4}\cdot S_{4}$$

**Figure 1.**
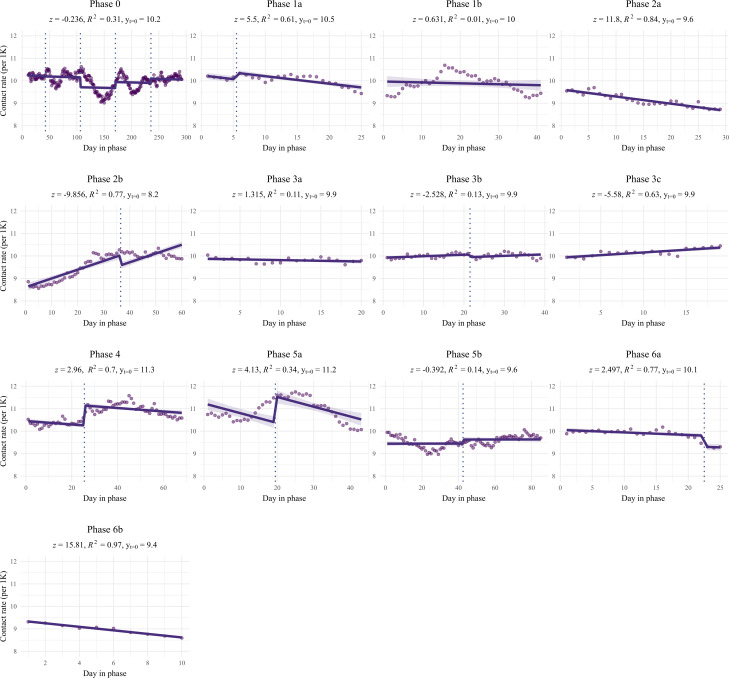
Linear models of health care consumption per phase, shown by day in phase and per 1000 registered pregnant patients. The *z* scores represent the comparison of the slope of each pandemic phase as compared to the slope of the prepandemic phase (Phase 0) along with their significance. As the prediction models for health care consumption were adjusted for seasonality, the linear regression lines may shift within a phase; these shifts are marked by vertical dashed lines at the seasonal transitions. The distributions of the x-axes are not equal for each phase, as phases did not consist of an equal number of days.

In this equation, Y_T_ represents the contact rate at time T, P the phase being modelled (ranging from 0 to 6b), β_0_ the intercept value at the start (*t*=0) of phase P, β_1_ the slope of the linear model during phase P, and T the time variables (in days within phase P). β_2_, β_3_, and β_4_ are the seasonal intercepts. S_1_, S_3_, S_4_ are dummy variables indicating the season (Winter, Spring, Summer, and Autumn), taking value 1 if the season is within the studied phase, and value 0 otherwise (see Figure 1). S_2_ was considered the baseline, always having value 0. This model captures the variation in the health care consumption during each phase, considering both the overall time trend (β_0_ + β_1_ ∙ T) and the seasonal effects introduced by the dummy variables β_2_ ∙ S_1_+ β_3_ ∙ S_3_ + β_4_ ∙ S_4_, taking spring S_2_ as the baseline.

Beta coefficients (ie, intercept β_0_ and slope β_1_) were computed for each subphase. Intercept values and their SEs were calculated at the start of each subphase (*t*=0) to determine immediate effects at the start of a new subphase. In addition, slopes and their SEs were computed to model and assess the trend in health care consumption within each subphase, where a negative slope indicated a decrease, and a positive slope indicated an increase in health care consumption. To compare intercept values and slopes of pandemic subphases to the prepandemic baseline, *z*-scores of the β-coefficients and their accompanying *P* values were calculated. Coefficients of determination (*R*^2^) and adjusted *R*^2^ were calculated to describe the proportion of variance that was explained by the independent variables within each model. Differences between a pandemic subphase and the prepandemic baseline were considered significant if the *P* value of the *z* score of either the intercept or the slope was <.05.

#### Analysis of Pregnancy-Relevant Symptoms and Diagnoses

The changes in the most prevalent pregnancy-related symptoms and diagnoses were examined by identifying the 10 most frequently registered ICPC codes from the created list of pregnancy-relevant ICPC codes (ie, symptoms and diagnoses) within the studied population during the studied period. Focusing on the most prevalent conditions allowed for a structured analysis of variation over time while maintaining analytical feasibility. The number of ICPC code registrations was calculated by normalizing the number of ICPC code registrations per 1000 contacts. To assess whether these proportions differed substantially between each pandemic subphase and the prepandemic baseline, statistical analysis through Fisher Exact tests for each specific ICPC code was conducted.

#### Analysis of Type of Contact

The type of contact was visualized as a percentage of the total number of contacts within each phase. Visualization was done using a centered moving average of 3 weeks and smoothed using Locally Estimated Scatterplot Smoothing. This nonparametric smoothing technique uses locally weighted polynomial regression to fit a smooth curve through points in a scatter plot, providing a visual trend of the data without assuming a specific parametric model.

### Ethical Considerations

Ethical approval for the collection and analysis of COVID-19 EHR data was conducted by the Medical Ethics Committee of the UMCG (2020/309). In this study, no patients were directly involved, and thus, this research was ruled not to be subject to the Medical Research Involving Human Subjects Act (Article 9, paragraph 2, EU-GDPR 2016/679). The EHR data were anonymized and deidentified before being made available to the researchers.

## Results

### Population Characteristics

A total of 975,545 contacts of 78,941 women in the reproductive age (20‐45 y) were screened for pregnancy status, of which 10,985 women (13.9%) were labelled as pregnant and had 39,023 contacts with the GP (see Table 2). Pregnant women had a mean age of 30.4 (SD 4.6) years during the total studied period. These women had an average of 2.8 (SD 2.6) contacts in 2019, 2.8 (SD 2.6) in 2020, and 2.9 (SD 2.8) in 2021.

**Table 2.** Demographic characteristics of the study population of pregnant women per year (2019‐2021; n=10,985).

	Total population^a^ (N=10,985)	Population in 2019 (n=4414)	Population in 2020 (n=4553)	Population in 2021 (n=4836)	*P* value
Pregnant women, n	10,985	4414	4553	4836	<.001
Age (years), mean (SD)	30.4 (4.6)	30.5 (4.6)	30.3 (4.6)	30.5 (4.5)	≥.99
Total contacts, n	39,023	12,384	12,686	13,953	<.001
Number of contacts per patient, mean (SD)	3.6 (3.5)	2.8 (2.6)	2.8 (2.6)	2.9 (2.8)	.99
Number of ICPC^b^ code registrations per contact, mean (SD)	1.13 (0.38)	1.14 (0.39)	1.13 (0.37)	1.12 (0.37)	≥.99

a Populations partly overlap in the 3 studied years.

b ICPC: International Classification of Primary Care.

### Changes in Health Care Consumption

Health care-seeking behavior differed significantly in each pandemic subphase compared to Phase 0 (see Figure 1 and Table 3). At the onset of the pandemic (Phase 1a), an immediate significant increase in health care consumption was observed (β_0_=10.54; *P=*.04), evident from the elevated intercept value compared to Phase 0. During Phase 1a, there was a substantial and significant decrease in health care consumption following the initial peak intercept value (β_1_=–0.03; *P<*.001), indicating reduced contacts with the GP at the beginning of the pandemic. In Phase 1b, an immediate effect was noted, with a significantly lower intercept value (β_0_=9.97, *P<*.001). However, the subsequent period did not significantly differ from Phase 0, indicating consistently lower health care consumption during Phase 1b, though this model was unable to explain most of the variance (*R*^2^=0.02).

**Table 3.** Interrupted time series analysis of health care consumption for the different pandemic subphases (2020 and 2021) compared to the prepandemic year 2019 (phase 0) of the total studied population of pregnant women, showing intercepts (β_0_) with SE, slopes with SEs (β_1_), *z* scores, and *P* values for intercepts.

Phase	Intercept of the linear model			Slope of the linear model			Model’s goodness of fit	
	Intercept β_0_ (SE)	*z* score, intercepts	*P* value, intercepts	Slope β_1_ (SE)	*z* score, slopes	*P* value, slopes	*R* ^2^	Adjusted *R*^2^
0 (baseline)	10.22 (0.04)	—^a^	—	−0.00 (0.00)	—	—	0.31	0.30
1a	10.54 (0.10)	–4.10	.04	–0.03 (0.00)	5.50	<.001	0.61	0.57
1b	9.97 (0.13)	0.97	<.001	–0.00 (0.01)	0.63	.53	0.02	0.01
2a	9.62 (0.05)	7.83	.33	−0.03 (0.00)	11.80	<.001	0.85	0.84
2b	8.16 (0.20)	9.33	<.001	0.04 (0.00)	−9.86	<.001	0.77	0.76
3a	9.88 (0.05)	3.31	<.001	−0.01 (0.00)	1.32	.19	0.11	0.06
3b	9.92 (0.04)	3.05	.001	0.01 (0.00)	−2.53	.01	0.13	0.08
3c	9.92 (0.05)	2.74	.002	0.02 (0.00)	−5.58	<.001	0.63	0.61
4	11.34 (0.11)	−10.13	.006	−0.01 (0.00)	2.96	.003	0.70	0.69
5a	11.23 (0.14)	−7.61	<.001	−0.04 (0.01)	4.13	<.001	0.34	0.31
5b	9.62 (0.14)	3.33	.001	0.00 (0.00)	−0.39	.70	0.14	0.11
6a	10.06 (0.06)	0.59	.56	−0.01 (0.00)	2.50	.01	0.77	0.75
6b	9.41 (0.03)	13.26	<.001	−0.08 (0.01)	15.81	<.001	0.97	0.97

a Not applicable.

The start of Phase 2a did not significantly differ from Phase 0 (β_0_=9.62; *P=*.33). Nevertheless, a significant negative slope indicated a sustained decline in health care consumption during this phase (β_1_=–0.03; *P<*.001). Phase 2b marked the lowest intercept value in health care consumption in the studied period (β_0_=8.16; *P<*.001). Subsequently, there was a significant increase in health care consumption during Phase 2b (β_1_=0.04; *P<*.001).

In Phase 3, all subphases (3a, 3b, and 3c) exhibited significantly lower intercept values (respectively, β_0_=9.88, *P<*.001; β_0_=9.92, *P*=.001; and β_0_=9.92, *P*=.002). Health care consumption remained stable during Phase 3a (β_1_=–0.01; *P*=.19), while significant increases occurred during Phases 3b and 3c (β_1_=0.01, *P=*.01 and β_1_=0.02, *P*<.001, respectively), though the models of Phase 3a and 3b were unable to explain most of the variance (*R*^2^=0.11, and *R*^2^=0.13, respectively).

The onset of Phase 4 marked the highest intercept value since the beginning of 2019 (β_0_=11.34; *P=*.006). However, health care consumption significantly declined during Phase 4 (β_1_=–0.01; *P=*.003).

Phase 5a began with a significantly higher intercept value (β_0_=11.23; *P<*.001). During Phase 5a, health care consumption declined significantly (β_1_=–0.04*; P<*.001). This decline led to a significantly lower intercept value at the beginning of Phase 5b (β_0_=9.62; *P=*.001), with health care consumption remaining relatively stable during Phase 5b. Phase 6a initiated with an intercept value comparable to Phase 0 (β_0_=10.06; *P=*.56). However, health care consumption declined significantly during Phase 6a (β_1_=–0.01; *P=*.01), resulting in a significantly lower intercept value of Phase 6b (β_0_=9.41; *P<*.001), followed by a further significant decline during Phase 6b (β_1_=–0.08; *P<*.001).

### Pregnancy-Relevant Symptoms and Diagnoses

#### Most Frequent Registered Symptoms and Diagnoses

The top 10 pregnancy-relevant symptoms and diagnoses (ie, ICPC codes) presented by pregnant women to their GP during the studied period were examined (see Multimedia Appendix 6). These symptoms and diagnoses were (1) pregnancy confirmed (ICPC W78), (2) cystitis or other urine infection (U71), (3) vomiting or nausea of pregnancy (W05), (4) abortion spontaneous (W82), (5) unwanted pregnancy confirmed (W79) (6) frequent or urgent urination, (U02), (7) urogenital candidiasis (X72), (8) GDM (W84.02), (9) other localized abdominal pain (D06), and (10) constipation (D12). Contacts related to these top 10 symptoms and diagnosis covered 65.6% (n=25,587) of all contacts of pregnant women during the studied period.

#### Changes in Pregnancy-Relevant Symptoms and Diagnosis

The normalized and absolute number of contacts concerning the top 10 symptoms and diagnoses per phase were examined (Multimedia Appendix 6). Health care consumption concerning pregnancy-related symptoms and diagnoses changed statistically significant in multiple pandemic phases compared to Phase 0. Specifically, health care consumption for vomiting or nausea of pregnancy was significantly higher during Phase 2a, 2b, 3b, 3c, 4, 5b, and 6b compared to Phase 0. Conversely, confirmed pregnancy was significantly lower in Phase 1a, 1b, 4, and 6a compared to Phase 0. Confirmed unwanted pregnancy was significantly lower in Phase 2b, 3b, 4, 5a, 5b, and 6a compared to Phase 0. Spontaneous abortion was found to be significantly higher in Phase 2a and 3b compared to Phase 0 (*P=.*04 and *P*=.048, respectively). GDM was significantly higher in the Phase 2a, 4, 5a, and 5b compared to Phase 0.

Notably, among general health symptoms and diagnoses, health care consumption for localized abdominal pain did not change significantly during the pandemic phases compared to Phase 0. Constipation declined significantly in Phase 5a compared to Phase 0 (*P=*.02). Frequent or urgent urination declined significant in Phase 3c compared to Phase 0 (*P=*.02). Cystitis or other urine infection was significantly higher in Phase 5b and lower in Phase 6a compared to Phase 0 (*P<*.001 and *P*=.04, respectively). Urogenital candidiasis showed a significant increase in Phase 5b compared to Phase 0 (*P=*.002).

### Changes in Type of Contact

**Figure 2.**
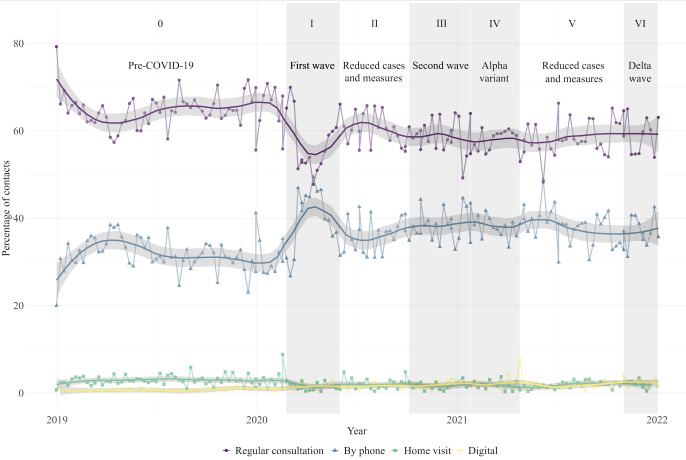
The proportion of types of contact for pregnant women across prepandemic and pandemic phases, with raw and smoothed percentages shown by contact date. Contact types were visualized as a percentage of the total number of contacts within each subphase, using a centered moving average of 3 weeks.

Figure 2 shows the percentages of type of contact (ie, regular consultations, telephone consultations, home visits, and digital consultations). During the initial pandemic phase, regular consultations declined, and telephone consultations increased. From Phase 2 until Phase 6, regular consultations increased, but did not reach the prepandemic baseline of 64.9% (n=9281). After the sharp increase in telephone consultations, there was a decline during the following pandemic phases. However, the percentage of telephone consultations remained higher than the prepandemic baseline of 31.7% (n=4529). Digital consultations remained comparable to the prepandemic baseline throughout the pandemic phases.

## Discussion

### Principal Findings

To the best of our knowledge, this is the first study examining pregnancy-relevant symptoms and diagnoses in GP practices in relation to the COVID-19 pandemic. By analyzing routine clinical EHR data registered by GPs, this study revealed statistically significant fluctuations in health care consumption among over 10,000 studied pregnant women during various pandemic phases, defined by infection rates and restrictive measures. In addition, this study demonstrated significant increases in GDM and vomiting or nausea of pregnancy, while contacts concerning confirmed pregnancies declined.

The deployed interrupted time-series analysis is a highly effective design for studying effects of interventions [21], such as the restrictive measures mandated by the government and changes in COVID-19 incidence rates during the pandemic phases. Furthermore, our combined method of selecting pregnant women led to the inclusion of a large sample of a wide range of pregnant women in the Netherlands. This comprehensive analysis of contacts, symptoms, and diagnoses of pregnant women provided valuable insights into prenatal GP care and changes in health care–seeking behavior of these vulnerable women during the COVID-19 pandemic.

### Population Characteristics

Our study showed a statistically significant increase in the number of pregnancies registered at the GP practice and absolute number of contacts in 2021, which is in accordance with national birth rates [22]. The mean age of the pregnant women studied (30.4 y, SD 4.6) is slightly lower than the mean maternal age at the date of delivery of the total Dutch population (31.6 y). Our study illustrated that, if pregnant women contacted their GP, the mean number of contacts in the prepandemic year 2019 was 2.8 (SD 2.6) and remained comparable in the pandemic years 2020 and 2021.

### Health Care Consumption

We show primarily declining health care consumption during the initial phases of the pandemic. The hesitation of pregnant women to seek GP care may have been influenced by the lack of knowledge about the consequences of a COVID-19 infection on both mother and fetus along with related news messages, fear of contamination, societal recommendations to stay home, or limited child care availability [23-25]. The declining trend also aligns with findings from other studies among the general population, which observed a pandemic-induced decrease in primary health care consumption initially [1-5,26]. Recent research has further highlighted a more pronounced decline in health care–seeking behavior among women compared with men during these initial pandemic phases, indicating that women may have been particularly hesitant to seek medical care amidst the pandemic [27]. Furthermore, a previously published systematic review revealed notable shifts in pregnant women’s health care–seeking behavior in other health care settings such as obstetric hospital and prenatal clinic care, including a substantial decline in both routine and unscheduled prenatal care visits at their maternal health care providers [28]. This decline may indicate that important prenatal care was missed during this period, potentially resulting in delayed adverse effects on mother, fetus, and neonate. Future research should elaborate on these long-term consequences.

After reaching the lowest point of GP health care consumption in the studied period (2020‐2021) in July 2020, health care consumption mainly increased until January 2021 and remained statistically significantly higher than the prepandemic level until mid 2021, suggesting a rebound after the initial decline. The observed increase mid-2020 may be explained by the declining infection rates and the gradual reopening of society. Furthermore, the increasing health care consumption among pregnant women may be indicative of an increased demand for GP health care, potentially compensating for earlier missed care. Other research illustrated similar fluctuating trends in health care–seeking behavior worldwide during these pandemic phases [1,27,29]. Despite this temporary rebound of health care consumption, the World Health Organisation identified a continued disruption of access to prenatal and primary health care services worldwide during this period [29].

Notably, from mid-2021 until the end of the year, a recurrent steep decline in health care consumption was observed, despite declining infection rates, minimal restrictions, and the gradual reopening of society. Possibly, pregnant women sought care with their primary care midwife again. However, the hesitation in contacting the GP may also be explained by news messaging highlighting an increased risk of serious illness from COVID-19 infection for pregnant women during this period, which was based on related published research [30]. This implies a negative impact of the rise of the Delta variant and reintroduced lockdown measures on prenatal health care, potentially resulting in recurrent missed prenatal care.

Overall, the declining trends in health care consumption during the start and end phases of the pandemic among pregnant women suggest a substantial impact on health care consumption during the pandemic, influenced by the COVID-19 incidence rates, restrictive measures, and news messaging related to COVID-19. Previous studies found that factors such as the fear of infection during health care contacts, limited understanding of the effects of COVID-19 on maternal and fetal health, and societal restrictions probably influenced this declining trend in health care consumption [1,23]. This could potentially lead to delayed adverse pregnancy outcomes, and physical and mental health problems [23,28]. Future studies should elaborate on these long-term consequences of the pandemic and on additional factors contributing to variation in health care consumption.

### Pregnancy-Relevant Symptoms and Diagnoses

Building upon these fluctuating trends in health care consumption, we also explored changes in presented pregnancy-relevant symptoms and diagnoses. We show that 83.8% (n=21,621) out of the top 10 most frequently registered symptoms and diagnoses were related to pregnancy, which largely aligns with prepandemic research among Dutch pregnant women in GP care [10]. Interestingly, most changes between pandemic phases and the prepandemic baseline were found in pregnancy-related symptoms and diagnoses, while fewer changes were observed in categories related to routine health care symptoms and diagnoses. Most symptoms and diagnoses consistently declined or consistently increased in frequency, independent of pandemic phases. This suggests that changes in frequency of presented symptoms and diagnoses were driven by the nature of the specific complaint or diagnosis, rather than being influenced by the prevailing measures or incidence rates during each phase.

We have underlined a noteworthy increase in health care consumption concerning GDM during different pandemic phases, consistent with previous research [31-34]. This might be attributed to a rise in pregnancy-related weight gain, as was shown by other studies [32,35]. We speculate that this consequential increase in GDM could be due to physical limitations, sedentary behavior, and emotional distress following the severely restrictive lockdown measures [35-37]. Therefore, pandemics can be a risk factor for developing GDM. This is an important finding given the results of a recent review underlining that GDM increases risks of adverse maternal and perinatal outcomes, such as hypertensive disorders of pregnancy, induction of labor, caesarean section, large-for-gestational-age neonates, preterm birth, and neonatal intensive care unit admission [38]. Furthermore, the increase in GDM may have contributed to the statistically significant increase in cystitis or other urine infection and urogenital candidiasis in some pandemic phases.

In addition, we speculate that the previously reported decreased physical behavior [37,39], increased sedentary behavior, and emotional distress may have also contributed to the observed increase of vomiting or nausea of pregnancy. Furthermore, previous reviews underlined a negative influence of the pandemic on eating behavior, influenced by emotions, mood, cravings, and environmental factors [40,41], which could have further worsened symptoms of vomiting and nausea. Nevertheless, no previous research was found regarding this increased complaint. Further research is needed to examine these notable increases in GDM and nausea or vomiting of pregnancy, and their impact on long-term health outcomes for both mother and child. This could enable the development of appropriate lifestyle interventions for pregnant women to be provided as part of routine prenatal GP care, with a positive impact on outcomes during future pandemics.

Furthermore, our study brought to light notable declines in the registration of confirmed wanted and unwanted pregnancies during various pandemic phases, despite consistent national birth rates in 2020, and even higher rates in 2021 [22]. This implicates a decline in women reporting pregnancies to their GP or a decline in routine contacts where these codes are registered, aiming to prevent strain on GP care for what may seem like trivial reasons for care. Consequently, an important proportion of pregnant women did not consult their GP and were, therefore, not included in the study. The pandemic led to changes in the organization of prenatal health care provision by maternal health care professionals (midwives and obstetricians) and GPs, and probably contributed to a decline in the number of pregnant women consulting their GP. Further studies should elaborate on these suggestions. Yet, contacting the GP about pregnancy is important, as GPs provide essential prenatal care, including health promotion, timely interventions for general health care problems, management of medication needs, and referrals [8,9,11,42]. Furthermore, women with unwanted pregnancies experience more psychosocial problems than those with wanted pregnancies and often rely on GPs for care, highlighting the importance of contacting the GP [43].

### Type of Contact

Considering the types of contact, our study showed a decrease in the proportion of regular physical contacts at the onset of the pandemic among pregnant women, accompanied by an increase of phone contacts. In line with previous research among the general population, while the proportion of regular physical contacts increased during subsequent phases, it did not return to the prepandemic levels during the studied years [1,28]. The pandemic accelerated the adoption of telehealth technologies, mainly through telephone contacts, in prenatal health care, facilitating more individualized and efficient health care [44,45]. A potential long-term shift toward a greater reliance on telehealth among pregnant women and GPs seems to have taken place. Nevertheless, further research should elaborate on whether this shift persisted postpandemic.

### Limitations

There are also some limitations to this study. EHR data are not specifically intended for scientific purposes, but rather for informing other physicians. Therefore, important information could be overlooked when assessing presented symptoms and diagnoses. Despite this, it is imperative to analyze such data as they represent one of the few available sources that provide real time, longitudinal insights into patient’s health care–seeking behavior. In addition, we could not assess the impact of other external factors, such as COVID-19-related news messaging and changes in other health care provision by maternal health care providers on health care consumption, due to limited data availability and the scope of the study design. However, these factors may have contributed to the limited explanation of the variation in health care consumption by the modeled independent variables across certain subphases. Future research should explore the influence of such factors. Furthermore, the endpoint of our data collection in 2021 limits our ability to assess the long-term effects or follow-up of pregnancy outcomes. To address this limitation, we recommend linking GP registration data with birth registration data over a longer term to comprehensively track the entire course of pregnancy and its outcomes. Finally, while the data are a reliable reflection of the Dutch general population, it should be noted that the results may not be fully generalizable to countries with different health care systems for pregnant women. Despite these constraints, it remains essential to analyze real-life EHR data to understand immediate trends and outcomes within the context of the health care system.

### Conclusions

In conclusion, this study highlights the impact of the pandemic on pregnant women’s health care–seeking behavior among more than 10,000 studied pregnant women. By analyzing routine clinical EHR data registered by GPs, we have underlined declining health care consumption trends during the initial and end phases of the pandemic (2020‐2021), the increase in pregnancy-related symptoms and diagnosis, such as GDM and nausea or vomiting of pregnancy, and the (temporary) adoption of a renewed way of providing health care to pregnant women through telehealth. Changes in frequency of presented symptoms and diagnoses were likely driven by the nature of the specific complaint or diagnosis. Physical limitations, sedentary behavior, and emotional distress following the severely restrictive lockdown measures may have attributed to the increased presentation of certain conditions, such as GDM and nausea or vomiting of pregnancy.

Given the potential adverse long-term effects of GDM on mother, fetus, and neonate, the urgent need for enhanced public health strategies within GP practices for pregnant women is undeniable. It is crucial for health care policymakers, providers, and pregnant women to recognize the risks associated with avoiding GP health care during this vulnerable period and work collaboratively to ensure safe and high-quality care during future pandemics, but also in routine health care settings. Implementing proactive measures to address these challenges could enhance the protection of the health and well-being of expectant mothers, fetuses, and neonates during times of crisis and beyond.

## Supplementary material

10.2196/64831 Multimedia Appendix 1 Used International Classification of Primary Care (ICPC) codes for defining pregnant and postpartum women.

10.2196/64831 Multimedia Appendix 2 Regular expression for textual analysis of general practitioner (GP) texts for defining pregnancy.

10.2196/64831 Multimedia Appendix 3 Assumptions met during manual labelling of general practitioner (GP) texts for defining pregnancy.

10.2196/64831 Multimedia Appendix 4 Flowchart of patient inclusion and exclusion.

10.2196/64831 Multimedia Appendix 5 Pregnancy-relevant International Classification of Primary Care (ICPC) codes used for the analysis of symptoms and diagnoses.

10.2196/64831 Multimedia Appendix 6 Distribution of the top 10 pregnancy-related International Classification of Primary Care (ICPC) code registrations.

## Abbreviations

AHON: Academic General Practitioner Development Network
EHR: electronic health record
GDM: gestational diabetes mellitus
GP: general practitioner
ICPC: International Classification of Primary Care
UMCG: University Medical Center Groningen
