# Supplementary material for: Impact of COVID-19 on Dutch General Practitioner Prenatal Primary Care: Retrospective, Observational Cohort Study Using an Interrupted Time-Series Approach
Source: JMIR Pediatr Parent. 2025 May 27;8:e64831. doi: 10.2196/64831 (PMC12133074; doi:10.2196/64831)
Supplement: Multimedia Appendix 1 [file pediatrics-v8-e64831-s001.docx]

## Multimedia Appendix I

**Supplementary table 1.** Used ICPC codes for defining pregnant and postpartum women.

| **ICPC code** | **ICPC title** |
| --- | --- |
|  |  |
| W03 | Antepartum bleeding |
| W05 | Pregnancy vomiting/nausea |
| W17 | Post-partum bleeding |
| W18 | Post-partum symptom/complaint other |
| W19 | Breast/lactation symptom/complaint |
| W20 | Other symptoms/complaints of breasts during pregnancy/postpartum |
| W27 | Fear complications of pregnancy |
| W29 | Pregnancy symptom/complaint other |
| W70 | Puerperal infection/sepsis |
| W71 | Infection complicating pregnancy |
| W77 | Pregnancy complicating non-obstetric factor |
| W78 | Pregnancy |
| W79 | Unwanted pregnancy |
| W81 | Toxaemia of pregnancy |
| W82 | Abortion spontaneous |
| W84 | Pregnancy high risk |
| W90 | Uncomplicate labour/delivery livebirth |
| W91 | Uncomplicate labour/delivery still |
| W92 | Complicate labour/ delivery livebirth |
| W93 | Complicate labour/delivery stillbirth |
| W94 | Puerperal mastitis |
| W95 | Breast disorder in pregnancy other |
| W96 | Complications of puerperium other |
| W99 | Disorder pregnancy/delivery, other |
