# Supplementary material for: Impact of COVID-19 on Dutch General Practitioner Prenatal Primary Care: Retrospective, Observational Cohort Study Using an Interrupted Time-Series Approach
Source: JMIR Pediatr Parent. 2025 May 27;8:e64831. doi: 10.2196/64831 (PMC12133074; doi:10.2196/64831)
Supplement: Multimedia Appendix 2 [file pediatrics-v8-e64831-s002.docx]

## Multimedia Appendix II

**Supplementary table 2.** Preliminary search terms and regular expressions for textual analysis of (Dutch) SOEP texts for defining pregnancy (search terms by author MH, general practitioner).^a^

| **Search terms in Dutch and English** | **Used regular expressions** |
| --- | --- |
|  |  |
| Zwanger/zwangerschap (pregnant/pregnancy) | zwa?ngerschap\|prill?e?.*zwangers?c?h?a?p? |
| Negatieve zwangerschapstest (Negative pregnancy test) | ?<! (negatieve) )zwangersch\|(?<!(niet\|geen))zwa?nger\|zwangerschaps? ?test(?!.*negatief) |
| Graviditeit (graviditas) | gra[vf]ida\|gra[vf]iditeit |
| Para (*n*) | para(?!\\D) |
| Bevalling/ bevallen (childbirth/ to give birth) | be[vf]all?en\|thuis ?bevalling |
| Geboorte/ geboren (birth/ born) | geboo?rt?e?n?\| |
| Baby, neonaat (baby/ neonate) | neonaa?t\|ongeboren.* baby |
| Prematuur (premature) | prematuur |
| Foetus (fetus) | f(o\|eu\|oe\|e)tus\|f(oe\|e\|eu)tale |
| Misselijkheid, hyperemesis gravidarum (nausea) | h[y\|ie]per(e\|ae\|ea)mesis\|gr[vf]idaa?ru[mn] |
| Pre-, peri-, postnatale zorg (pre-, peri-, postnatal care) | pre(-?\| ?)nata\|post(-?\| ?nata\|peri(-?\| ?)nata |
| Kraam (-week/-verzorging/-hulp/-weken/ -periode/miskraam) (postpartum week/ care/ help/ weeks/ period/ miscarriage) | kraam |
| Borstvoeding/ lactatie/ mastitis (breastfeeding/ lactation) | borst( ?\|ge)[vf]oe(d\|t\|dt)\|la[ck]t[ae]a?(tie\|ren)\| mae?st(i\|ie\|y)t(i\|e\|y)s |
| Verloskundige (midwife) | verlosk |
| A terme datum/ datum uitgerekend (estimated due date) | aterme. *datum\|datum.*uitgerekend |
| Missed abortion | missed ?abortion |
| In verwachting (expecting) | in verwachting |
| Vruchtwater (amniotic fluid) | vruchtwater |
| Placenta | placenta |
| Keizersnede (caesarean section) | k(ei\|ij)zersnede\|sectio |
| Gemelli (twins) | gemell?i |
| In stuit (breech) | in stuit |
| Kolven (pumping breast milk) | kolven |
| HELLP | HELLP |

^a^SOEP = Subjective, Objective, Evaluation, Plan.
