# Supplementary material for: Impact of COVID-19 on Dutch General Practitioner Prenatal Primary Care: Retrospective, Observational Cohort Study Using an Interrupted Time-Series Approach
Source: JMIR Pediatr Parent. 2025 May 27;8:e64831. doi: 10.2196/64831 (PMC12133074; doi:10.2196/64831)
Supplement: Multimedia Appendix 3 [file pediatrics-v8-e64831-s003.docx]

## Multimedia Appendix III

Selection of most prevalent reasons to consider a pregnancy status as positive or negative, concerning the manual assessment of physicians notes of contacts between a patient and a general practitioner.

### Cases Considered as Positive Pregnancy Status

- Typing errors or abbreviated writing, e.g., “zwschp” or “zw schap” meaning “zwangerschap” (“pregnancy”)
- Texts indicating the consideration an abortion, e.g. “denkt aan abortus” (“considers abortion”)
- Texts related to blood loss combined with other pregnancy-related topics, e.g., contact with primary care midwife
- Reporting of the pregnancy term, e.g. “20+5 weeks”
- Reporting of the unborn child, e.g., “gedachten jegens ongeboren kind” (“thoughts toward unborn child”)

### Cases Considered as Negative Pregnancy Status

- Cases of doubt about pregnancy, e.g., “mogelijk zwanger” (“possibly pregnant”)
- Reporting of delivery of child
- Reporting of postpartum subjects, e.g., maternity period, breastfeeding pumping
- Reporting of fertility trajectory, e.g., “start traject fert.” (“starting fertility trajectory)
