# Supplementary figures and images for: Impact of COVID-19 on Dutch General Practitioner Prenatal Primary Care: Retrospective, Observational Cohort Study Using an Interrupted Time-Series Approach
Source: JMIR Pediatr Parent. 2025 May 27;8:e64831. doi: 10.2196/64831 (PMC12133074; doi:10.2196/64831)

## Multimedia Appendix IV


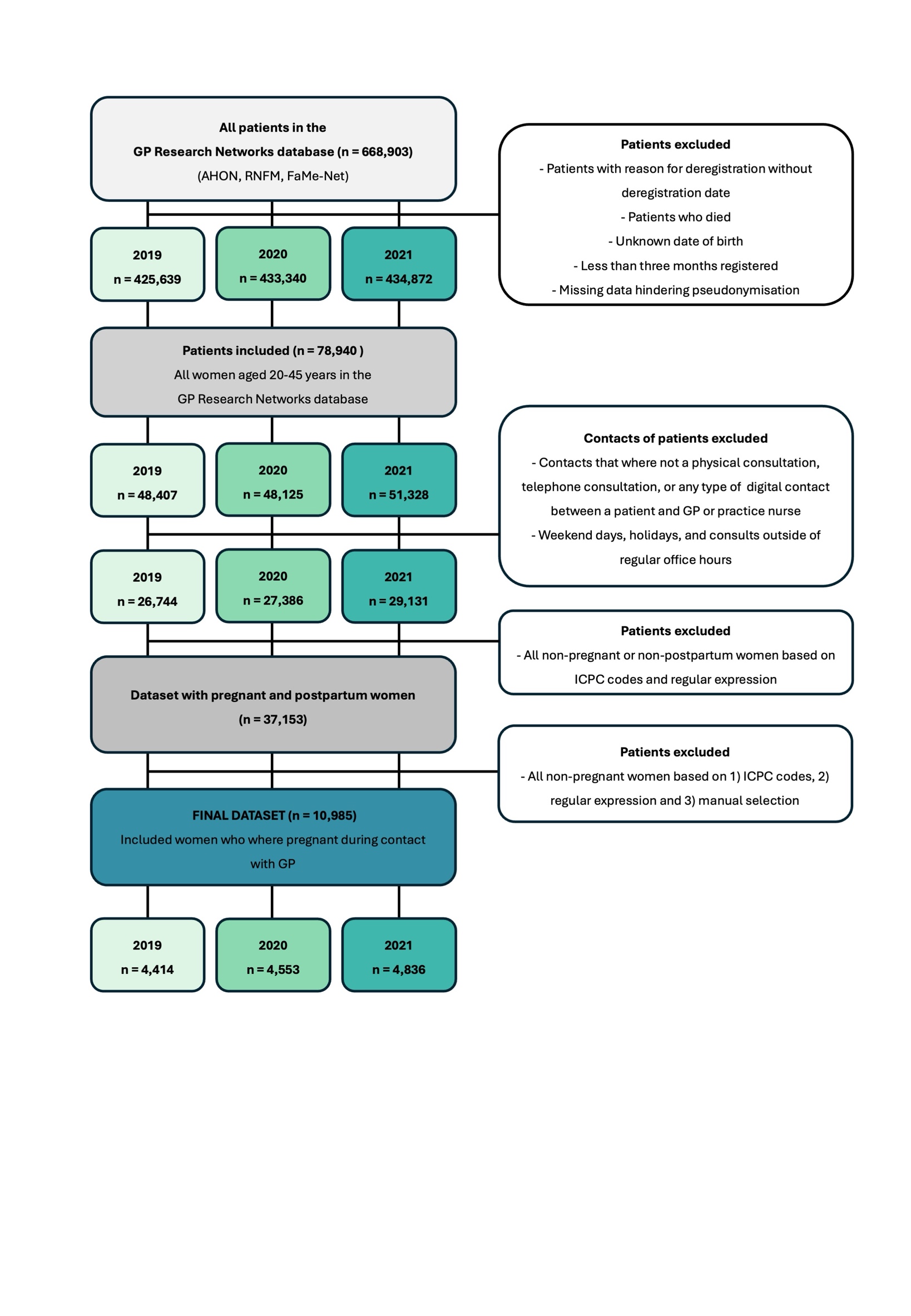


**Supplementary figure 1.** Flowchart of patient inclusion and exclusion

Supplement: Multimedia Appendix 4 [file pediatrics-v8-e64831-s004.docx]
