# Supplementary material for: Impact of COVID-19 on Dutch General Practitioner Prenatal Primary Care: Retrospective, Observational Cohort Study Using an Interrupted Time-Series Approach
Source: JMIR Pediatr Parent. 2025 May 27;8:e64831. doi: 10.2196/64831 (PMC12133074; doi:10.2196/64831)
Supplement: Multimedia Appendix 5 [file pediatrics-v8-e64831-s005.docx]

## Multimedia Appendix V

**Supplementary table 3.** The list of pregnancy-relevant ICPC codes used for the analysis of pregnancy-relevant symptoms and diagnoses.

| **ICPC code** | **Description of complaint or diagnosis** |
| --- | --- |
|  |  |
| A04 | General weakness/tiredness |
| A09.00 A09.01, A09.02 | Sweating problems |
| A97 | No disease |
| A99 | Other general/unspecified diseases |
| D02 | Stomach pain/ache |
| D03 | Heartburn |
| D06 | Other localized abdominal pain |
| D08 | Flatulence/gas pain/belching |
| D09 | Nausea |
| D12 | Constipation |
| D84.00, D84.02, D84.03, D84.06 | Disease of oesophagus |
| K06 | Prominent veins |
| K07 | Swollen ankles/oedema |
| K25 | Fear of hypertension |
| K85 | Elevated blood pressure |
| K86 | Uncomplicated hypertension |
| K95 | Varicose veins of legs |
| K96 | Haemorrhoids |
| L02 | Back symptoms/complaints |
| L03 | Low back complaint excluding radiation |
| L86 | Lumbar disc lesion/radiation |
| N01 | Headache |
| N02 | Tension headache |
| N05 | Tingling fingers/feet/toes |
| N16 | Disturbance of smell/taste |
| N17.00, N17.01, N17.02 | Vertigo/dizziness |
| P01 | Feeling anxious/nervous/tense |
| P03 | Feeling depressed |
| P06 | Disturbances of sleep/insomnia |
| P29 | Other psychological symptoms/complaints |
| P74 | Anxiety disorder/anxiety state |
| P76 | Depressive disorder |
| R02 | Shortness of breath/dyspnoea |
| R04.00, R04.01 | Other breathing problems |
| R05 | Cough |
| R74 | Upper respiratory infection acute |
| R97 | Hay fever/allergic rhinitis |
| S02 | Pruritis |
| S23 | Baldness/losing hair |
| S74 | Dermatophytosis |
| S88 | Contact dermatitis/other eczema |
| T86 | Hypothyroidism/myxoedema |
| T87 | Hypoglycaemia |
| T88 | Renal glucosuria |
| T90.00, T90.01, T90.02 | Diabetes mellitus |
| U01 | Painful urination |
| U02 | Frequent/urgent urination |
| U05 | Other urination problems |
| U07 | Other complaints of urine |
| U13 | Other symptoms/complaints bladder |
| U70 | Pyelonephritis/pyelitis acute |
| U71.00, U71.01, U71.02 | Cystitis/other urine infection NOS |
| U72 | Urethritis nonspecific |
| W05 | Vomiting/nausea of pregnancy |
| W20 | Other symptoms/complaints breast in pregnancy |
| W27 | Fear complications of pregnancy |
| W28.00, W28.01 | Limited function/disability |
| W29.00, W29.01, W29.02 | Other symptoms pregnancy/family planning |
| W71 | Other infectious conditions |
| W73 | Benign neoplasm |
| W75 | Injuries complicating pregnancy |
| W76 | Congenital anomalies of mother |
| W77.00, W77.01, W77.02, W77.03 | Other non-obstetrical conditions/diseases affecting pregnancy, childbirth, and puerperium |
| W78 | Pregnancy confirmed |
| W79 | Unwanted pregnancy confirmed |
| W80 | Ectopic pregnancy |
| W81.00, W81.01, W81.02, W81.03 | Toxaemia (pre)eclampsia |
| W82 | Abortion spontaneous |
| W83 | Abortion induced |
| W84.00, W84.01, W84.02, W84.03, W84.04, W84.05, W84.06, W84.07, W84.08 | Pregnancy high risk  (W84.02, Gestational diabetes) |
| X14 | Vaginal discharge |
| X18 | Breast pain |
| X21 | Other symptoms/complaints breast |
| X72 | Urogenital candidiasis proven |
