# Supplementary material for: Impact of COVID-19 on Dutch General Practitioner Prenatal Primary Care: Retrospective, Observational Cohort Study Using an Interrupted Time-Series Approach
Source: JMIR Pediatr Parent. 2025 May 27;8:e64831. doi: 10.2196/64831 (PMC12133074; doi:10.2196/64831)
Supplement: Multimedia Appendix 6 [file pediatrics-v8-e64831-s006.docx]

## Multimedia Appendix VI

**Supplementary table 4.** The proportion and absolute number of registered ICPC code registrations per pregnancy-relevant ICPC code, displayed per 1,000 contacts, for the pre-pandemic baseline (phase 0) and the pandemic phases (phase 1a – 6b) along with their *p* values.^a^

|  | Other localised abdominal pain | | Constipation | | | Frequent/ urgent urination | | | Cystitis/ other urine infection NOS | | | Vomiting/nausea of pregnancy | | | Pregnancy confirmed | | | Unwanted pregnancy confirmed | | | Abortion spontaneous | | | Gestational diabetes mellitus | | | Urogenital candidiasis proven | | |
| --- | --- | --- | --- | --- | --- | --- | --- | --- | --- | --- | --- | --- | --- | --- | --- | --- | --- | --- | --- | --- | --- | --- | --- | --- | --- | --- | --- | --- | --- |
| **Phase** | D06  (n) | *p* value | D12  (n) | *p* value | U02  (n) | | *p* value | U71  (n) | | *p* value | W05  (n) | | *p* value | W78  (n) | | *p* value | W79  (n) | | *p* value | W82  (n) | | *p* value | W84.02  (n) | | *p* value | X72  (n) | | *p* value |  |
|  |  |  |  |  |  | |  |  | |  |  | |  |  | |  |  | |  |  | |  |  | |  |  | |  |  |
| 0 | 13.4  (192) |  | 13.0  (186) |  | 21.0  (300) | |  | 45.4  (649) | |  | 36.7  (525) | |  | 434.3  (6210) | |  | 32.5  (465) | |  | 40.8  (584) | |  | 11.1  (158) | |  | 13.0  (186) | |  |  |
| 1a | 8.1  (10) | 0.117 | 12.9  (16) | 1.000 | 20.1  (25) | | 0.918 | 49.9  (62) | | 0.479 | 27.4  (34) | | 0.095 | 384.6  (478) | | **0.001** | 29.8  (37) | | 0.676 | 32.2  (40) | | 0.152 | 8.1  (40) | | 0.391 | 16.1  (20) | | 0.364 |  |
| 1b | 9.6  (20) | 0.177 | 11.0  (23) | 0.531 | 17.3  (36) | | 0.283 | 45.6  (95) | | 0.955 | 38.9  (81) | | 0.619 | 411.2  (857) | | **0.049** | 31.2  (65) | | 0.791 | 39.8  (83) | | 0.859 | 7.7  (16) | | 0.207 | 13.4  (28) | | 0.837 |  |
| 2a | 16.4  (22) | 0.388 | 9.0  (12) | 0.249 | 13.4  (18) | | 0.068 | 47.8  (64) | | 0.681 | 53.8  (72) | | **0.003** | 408.5  (547) | | 0.069 | 26.1  (35) | | 0.223 | 53.0  (71) | | **0.038** | 24.7  (33) | | **0.000** | 13.4  (18) | | 0.899 |  |
| 2b | 12.9  (38) | 0.930 | 11.6  (34) | 0.588 | 17.7  (52) | | 0.283 | 51.1  (150) | | 0.193 | 49.7  (146) | | **0.001** | 439.9  (1292) | | 0.581 | 21.8  (64) | | **0.002** | 48.7  (143) | | 0.056 | 8.9  (26) | | 0.325 | 13.6  (40) | | 0.789 |  |
| 3a | 10.0  (10) | 0.471 | 12.1  (12) | 1.000 | 16.1  (16) | | 0.356 | 35.1  (35) | | 0.153 | 39.2  (39) | | 0.664 | 436.8  (435) | | 0.895 | 23.1  (23) | | 0.112 | 47.2  (47) | | 0.323 | 11.0  (11) | | 1.000 | 11.0  (11) | | 0.770 |  |
| 3b | 15.2  (30) | 0.534 | 10.1  (35.5) | 0.334 | 16.7  (33) | | 0.235 | 49.0  (97) | | 0.456 | 50.5  (100) | | **0.004** | 428.0  (847) | | 0.611 | 19.7  (39) | | **0.001** | 50.5  (100) | | **0.048** | 12.1  (24) | | 0.648 | 16.2  (32) | | 0.251 |  |
| 3c | 19.4  (20) | 0.127 | 10.7  (11) | 0.667 | 32.9  (34) | | **0.015** | 34.9  (36) | | 0.119 | 50.3  (52) | | **0.034** | 456.0  (471) | | 0.183 | 31.0  (32) | | 0.856 | 46.5  (187) | | 0.373 | 14.5  (15) | | 0.286 | 15.5  (16) | | 0.479 |  |
| 4 | 12.5  (50) | 0.696 | 15.5  (62) | 0.216 | 21.5  (86) | | 0.804 | 44.3  (177) | | 0.796 | 53.4  (213) | | **<0.001** | 407.8  (1628) | | **0.003** | 23.3  (93) | | **0.002** | 46.8  (187) | | 0.099 | 19.0  (76) | | **<0.001** | 16.8  (67) | | 0.078 |  |
| 5a | 11.3  (28) | 0.444 | 7.7  (19) | **0.023** | 19.4  (48) | | 0.647 | 37.5  (93) | | 0.081 | 41.1  (102) | | 0.302 | 422.8  (1049) | | 0.292 | 21.0  (52) | | **0.002** | 43.9  (109) | | 0.477 | 17.3  (43) | | **0.012** | 10.9  (27) | | 0.437 |  |
| 5b | 12.8  (56) | 0.821 | 12.6  (55) | 0.878 | 18.5  (81) | | 0.329 | 58.8  (257) | | **<0.001** | 45.3  (198) | | **0.012** | 413.5  (1808) | | 0.016 | 20.4  (89) | | **<0.001** | 36.8  (161) | | 0.251 | 15.6  (68) | | **0.022** | 19.4  (85) | | **0.002** |  |
| 6a | 15.0  (20) | 0.620 | 14.2  (19) | 0.705 | 25.4  (34) | | 0.276 | 32.9  (44) | | **0.037** | 46.4  (62) | | 0.083 | 405.4  (542) | | **0.043** | 21.7  (29) | | **0.033** | 43.4  (58) | | 0.665 | 16.5  (22) | | 0.081 | 9.0  (12) | | 0.249 |  |
| 6b | 17.2  (16) | 0.308 | 16.1  (15) | 0.375 | 22.6  (21) | | 0.723 | 40.9  (38) | | 0.569 | 74.2  (69) | | **<0.001** | 394.6  (367) | | 0.018 | 23.7  (22) | | 0.149 | 36.6  (34) | | 0.606 | 17.2  (16) | | 0.108 | 17.2  (16) | | 0.297 |  |

^a^ ICPC, International Classification of Primary Care; NOS, not otherwise specified.
